# KG2Vec: A node2vec-based vectorization model for knowledge graph

**DOI:** 10.1371/journal.pone.0248552

**Published:** 2021-03-30

**Authors:** YueQun Wang, LiYan Dong, XiaoQuan Jiang, XinTao Ma, YongLi Li, Hao Zhang

**Affiliations:** 1 College of Computer Science and Technology, Jilin University, Changchun, China; 2 Key Laboratory of Symbolic Computation and Knowledge Engineering of Ministry of Education, Jilin University, Changchun, China; 3 School of Computer Science and Technology, Northeast Normal University, Changchun, China; Fuzhou University, CHINA

## Abstract

Since the word2vec model was proposed, many researchers have vectorized the data in the research field based on it. In the field of social network, the Node2Vec model improved on the basis of word2vec can vectorize nodes and edges in social networks, so as to carry out relevant research on social networks, such as link prediction, and community division. However, social network is a network with homogeneous structure. When dealing with heterogeneous networks such as knowledge graph, Node2Vec will lead to inaccurate prediction and unreasonable vector quantization data. Specifically, in the Node2Vec model, the walk strategy for homogeneous networks is not suitable for heterogeneous networks, because the latter has distinguishing features for nodes and edges. In this paper, a Heterogeneous Network vector representation method is proposed based on random walks and Node2Vec, called KG2vec (Heterogeneous Network to Vector) that solves problems related to the inadequate consideration of the full-text semantics and the contextual relations that are encountered by the traditional vector representation of the knowledge graph. First, the knowledge graph is reconstructed and a new random walk strategy is applied. Then, two training models and optimizing strategies are proposed, so that the contextual environment between entities and relations is obtained, semantically providing a full vector representation of the Heterogeneous Network. The experimental results show that the KG2VEC model solves the problem of insufficient context consideration and unsatisfactory results of one-to-many relationship in the vectorization process of the traditional knowledge graph. Our experiments show that KG2vec achieves better performance with higher accuracy than traditional methods.

## 1 Introduction

Nowadays we reach an era that everything can be embedded, called representation learning. In many research fields, embedding models are adopted to vectorize the research data. For instance, in the field of natural language processing (NLP) [1], by embedding the words into the vector representation, we can determine a word’s synonym, or estimate the accuracy of the translation; in the field of bioinformatics, protein chain [2] or transcription factor [3]can be regarded as a network. By embedding the proteins into vectors, we can determine whether a chain bond exists; as in social network, by embedding social entities, link prediction can be performed. Therefore, many researchers have developed various 2vec models tailored to fields, such as word2vec [4] in NLPs, and Node2Vec [5] in social networks.

Currently, most heterogeneous information networks (HIN) measure the similarity between points by aiming at making dot products of two nodes as large as possible in low-dimensional space. This method can only consider first-order proximity, which is also mentioned in node2vec. Compared with homogeneous information network, heterogeneous information network contains multiple relationships, where each relationship has different types of semantic information, and the distribution of relationship types is very uneven.

For heterogeneous networks (i.e. Knowledge graphs), a more advanced algorithm represents the nodes and links as triples (head, relation, and tail). In KGs, we often project an entity to a low-dimensional vector *h (or t)* with dimension *n*, by considering the entity to be a node, and representing the relations as operations between nodes [6]. Therefore, a relational scoring function can be defined as *fr(h*,*t)*, by minimizing the distance between *fr* and real *r* as the target. By iteratively updating *h*, the vector projection of r and *t* can be obtained.

**Fig 1:**
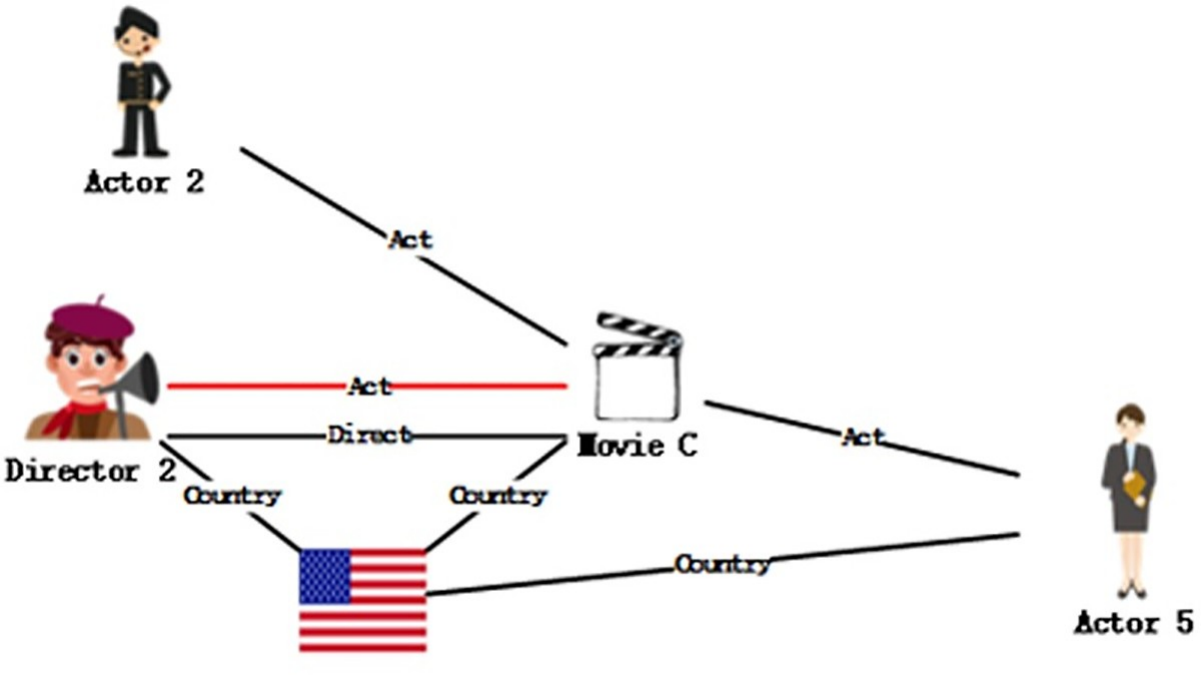
A subgraph of Fig 4.

The KG embedding algorithms like TransE [6], TransR [7] and TransG [8] are designed by this main idea. Although these algorithms are proved to be efficient in many scenarios, we notice that the trans-algorithms handle each triple with the same probability, lacking the emphasis as the 2vec models process the vectorization, resulting in unsatisfactory results. Taking the movie dataset as an example, the movie node A has relations with three actor nodes, one director node, and one country node, as shown in Fig 1. During the process of embedding, the influence of the country node and its relation is bound to be different from that of the director node.

**Fig 2:**
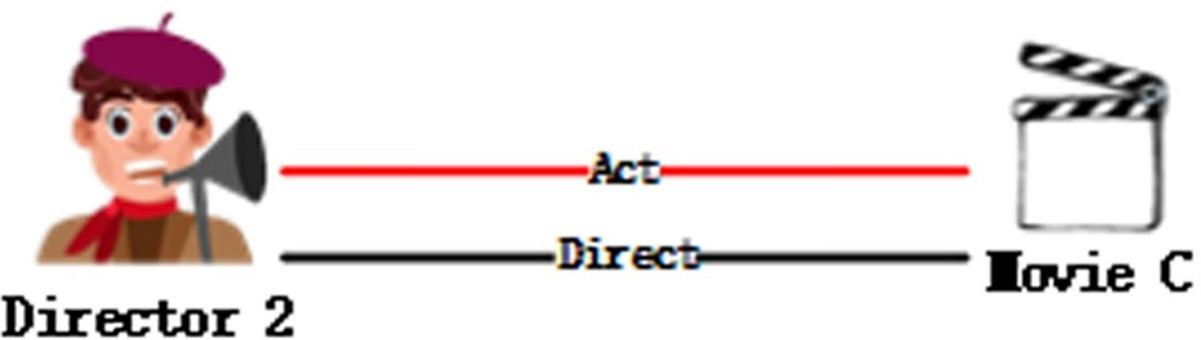
1-N relation in knowledge graph.

The trans-series algorithm can accurately predict 1-to-1 relation using triples, however, it has flaws in dealing with N-to-N, or 1-to-N. As shown in Fig 2, movie A is both directed and performed by B, meaning that there are two relation types between A and B, which cannot be trained using triples.

Taking 2vec and trans series algorithms into consideration, we aim to give certain weights to the triples in trans series by 2vec random walks [9], so that emphasis takes place during vectorization. However, the random walks in 2vec algorithms fail in heterogeneous networks. Besides, trans series using triples can neither give emphasis on different triples nor avoid the problem of multiple relations.

In this paper, the KG2Vec algorithm is proposed based on node2vec. Two challenges would appear if node2vec is directly applied to the vectorization of the heterogeneous networks: 1) heterogeneous networks are composed of entities (different types of nodes) and relations (different types of edges). For heterogeneous networks, the triple of form (head entity, relation, and tail entity) is the key to construct the node context. The node2vec algorithm neglects this key information, so that the quality of embedding is compromised. 2) Encountering the complexity of heterogeneous networks, the random walks strategy has to be adapted.

To solve the first problem, we propose to homogenize heterogeneous networks by abstracting the relation in heterogeneous networks to a new relation node and the node in heterogeneous networks to an entity node. Then we can use node2vec idea to train the reconstructed heterogeneous networks. However, the original random walks make no distinction between relation-type node and entity-type node, so the result is unsatisfactory.

Due to the natural advantages of heterogeneous network embedding (HNE) in application, which largely prevents the task performance from being attributed to effective data preprocessing and novel technical design, especially considering the various possible ways to build heterogeneous networks from the actual application data. As for heterogeneous networks, the 2vec random walks algorithm leading to the problem of inaccuracy embedding.

Thus, a new random walk strategy is proposed. The strategy learns the pattern “entity-relation-entity” as the main context. Therefore, we can find the most suitable context by this pattern in the reconstructed heterogeneous networks, as the input of this model.

Moreover, CBOW [10] and Skip-gram [11] are applied in the training process of KG2Vec, and the embedding of relation node and entity node are predicted. A parameter called node degree that adjusts the walk times is also introduced, in order to improve the quality and efficiency of the model.

The contributions of this paper are summarized as follows:

- The original heterogeneous networks are reconstructed, and an entity-relation topology is proposed.
- A new embedding method for heterogeneous networks is proposed and node2vec is improved. The new walk strategy is applied in the reconstructed heterogeneous networks. A node-degree parameter is also introduced to control the walk times.
- Two training models are proposed for heterogeneous networks: given relations, CBOW is used to predict the context entity; given entities, Skip-gram is used to predict the relation node.

## 2 Related work

This paper study the representation learning of heterogeneous information networks. In this chapter, the existing heterogeneous information network algorithm and the existing 2vec algorithm will be elaborated.

### 2.1 Heterogeneous network representation learning

A universal taxonomy is used for existing HNE algorithms with three categories based on their common objectives. The main challenge of instantiating on heterogeneous networks is the consideration of complex interactions regarding multi-typed links and higher order meta-paths. The HNE algorithm is mainly based on three ideas, including: Proximity-Preserving Methods, Message-Passing Methods and Relation-Learning Methods.

The goal of network embedding is to capture network topological information. This can be achieved by preserving different types of proximity among nodes. There are two major categories of proximity preserving methods in HNE: random walk-based approaches (inspired by DeepWalk) and first/second-order proximity based ones (inspired by LINE [12]). Metapath2vec [13] is one of the typical algorithms based on random walk, so does HIN2vec [14] algorithm. Metapath2vec utilizes the node paths traversed by meta-path guided random walks to model the context of a node regarding heterogeneous semantics. HIN2Vec considers the probability that there is a meta-path M between nodes *u* and *v*. SHNE [15] improves metapath2vec by incorporating additional node information. For such algorithms, it is generally necessary to establish a meta-path. However, one disadvantage of this path-based approach is that the path (pattern) needs to be specified (requiring more domain knowledge to do so). The first/second-order proximity-base algorithm includes PTE [16] algorithm, AspEm [17] algorithm, HEER algorithm and so on. PTE proposes to decompose a heterogeneous network into multiple bipartite networks, each of which describes one edge type. Its objective is the sum of log-likelihoods over all bipartite networks. AspEm assumes that each heterogeneous network has multiple aspects, and each aspect is defined as a subgraph of the network schema [18]. An incompatibility measure is proposed to select appropriate aspects for embedding learning. HEER extends PTE by considering typed closeness. Specifically, each edge type has an embedding.

For Message-Passing Methods, each node in a network can have attribute information represented as a feature vector. Message-passing methods aim to learn node embeddings based on the feature vector, by aggregating the information from u’s neighbors. In recent studies, Graph Neural Networks (GNNs) [19] are widely adopted to facilitate this aggregation/message-passing process. Different from regular GNNs, R-GCN [20] considers edge heterogeneity by learning multiple convolution matrices W’s, each corresponding to one edge type. During message passing, neighbors under the same edge type will be aggregated and normalized first. The node embedding is the output of the K-th layer. As the development of deep learning, in the field of graph feature representation, in addition to those traditional methods, deep learning methods have also been integrated, in order to embed node features. For instance, DKN [21] (Deep Knowledge-aware Network) embeds news titles through KG into vectorization, improving the accuracy. Besides, MKR [22], as well as in the recommendation systems, collaborates users and items into KG, adjusts RS and KG by taking the difference between the actual rate and the predicted rate as the loss function, thus regulating the user and item feature embedding.

For Relation-Learning Methods, we first highlight TransE and its variants (TransR [7] and TransG [8]) because they are simple and effective and can achieve the state-of-the-art performance in the majority of related tasks, especially in KGs with thousands of relations. TransE is one of the classic algorithms for KG embedding that was presented by Bordes et al in 2013. After this algorithm was presented, a series of algorithms were implemented, such as TransH [23], and TransG. Those traditional training methods introduce too many parameters when modelling the triples (head-relation-tail) in the knowledge base, leading to the low interpretability of the model and the overfitting problem during training. Meanwhile, TransE improved the cost function by introducing a reward and punishment mechanism, which maximized the prediction result by separating right from wrong as far as possible. Thus, it has remedied the problems related to complex training parameters and difficult expansions in traditional methods. TransE represents a relation as a vector r indicating the semantic translation from the head entity h to the tail entity t, aiming to satisfy the equation *t*−*h*≈*r* when triplet (h; r; t) holds.

Furthermore, Zheng Wang et al. propose TransH, which introduces two additional relation matrices compared to TransE related to the head and the tail. Instead of projecting the relations to another space, they use vectors to solve the difficulties of TransE in dealing with reflexive one-to-many many-to-one many-to-many relations. In addition, Guoling Ji et al improve TransE when encountering the link prediction problem using a method called TransD [24]. This algorithm defines the mapping matrices for every relation, thus improving the prediction accuracy and the computational complexity.

### 2.2 2Vec algorithm research

First, we review Word2vec and its extensions. Since Mikolov proposed the concept word embedding in his paper” Efficient Estimation of Word Representation in Vector Space” in 2013, the NLP field enters the world of “embedding”, such as Senternce2Vec [25], Doc2Vec [26], and Everthing2Vec. The word embedding is based on the assumption that the meaning of a word can be inferred from its context, proposing the word distributed representation. Compared with traditional One-hot Representation in NLP, which is high-dimensional and sparse, the word embedding trained by Word2Vec is both low-dimensional and dense. The main idea of Word2Vec is to make use of word context and yield richer semantic information. The current main applications are listed as follows:

1. The trained word embedding is used as the input feature to improve the existing system, for instance the input layer of neural networks such as sentiment analysis, part-of-speech tagging, natural language translation.
2. The word embedding is directly adopted from the perspective of linguistics, for instance, expressing the word similarity based on the distance of embeddings, and the query correlation. Word2vec employs a one-layer neural network (i.e. CBOW) to project the one-hot sparse word embedding to a n-dimensional dense vector.

Later, Word2vec has been transplanted in social networks. A.Grover designed a Node2vec model, which employs a weight parameter α, to control the random walks in Deepwalk, so that the resulting sequence is a combination of DFS and BFS [27]. This model makes use of Skip-gram in Word2vec as basis. The main contribution of Node2vec is considering a graph as a text, where the nodes in the graph can be represented by tokens in the text. Then Word2vec can be directly applied to yield vectors. However, the difference between graphs and texts lies in that texts are linear sequences, as graph has a more complex structure. The algorithm Deepwalk that was put forward before inspires Node2vec, which combines DFS and BFS as walk strategy to sample the nodes in graph. As Figure shows, BFS yields Local microscopic view, as DFS yields global macroscopic view. Node2vec introduces a heuristic approach 2nd-order random walks, namely defining random walks and two hyper parameters [28].

**Fig 3:**
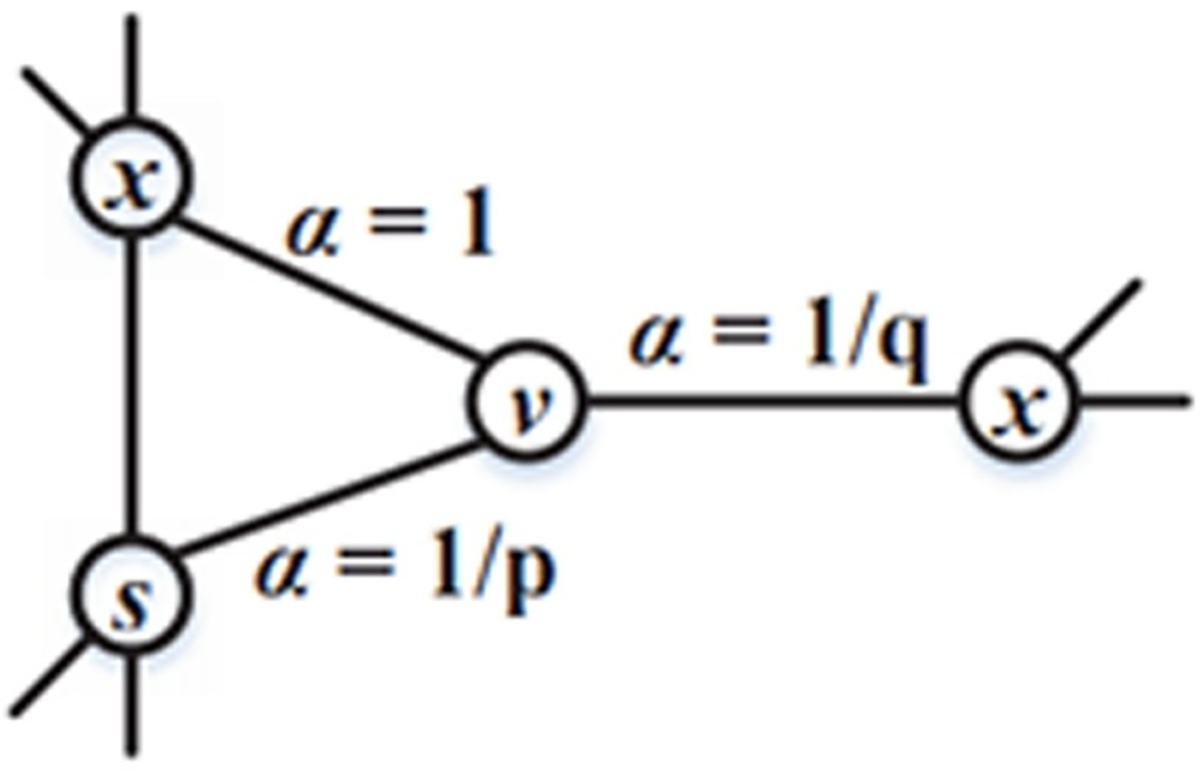
The transition probability process of Node2Vec.

Fig 3 shows the transition probability process of Node2Vec. The better the random walk is, the more appropriate context the algorithm finds, and the more efficient it is. Eq 1 is used to calculate the skip probability of Node2vec.

$$P(c_{i}=x|c_{i-1}=v)=\left\{ \begin{array}{c} \frac{\pi_{vx}}{Z}if\;(v,x)\in E\\ 0\;otherwise \end{array}\right.$$ (1)

where *π*_*vx*_ represents the skip probability between social nodes, and *z* is a normalization parameter.

Conversely, in Deepwalk, which is another embedding algorithm, the skip probability equals the weights that are labelled on the edges between nodes, such as *π*_*vx*_ = *W*_*vx*_ The bias parameter influences Node2vec in the way that it regularizes the random walk and balances the BFS and DFS, thereby equipping the under-predicted nodes with better context.

$$\alpha_{pq}(s,x)=\left\{ \begin{array}{c} \frac{1}{p}if\;d_{sx}=0\\ 1\;if\;d_{sx}=1\\ \frac{1}{q}if\;d_{sx}=2 \end{array}\right.$$ (2)

In Eq 2, *p* and *q* are used to adjust the walks, and *d*_*sx*_ represents the smallest distance between *s* and *x*, which has the largest value of 2. Supposing the current node is *v* and the last hop is *s*, the parameter *p* defines the probability of jumping back. It can be concluded that the higher the probability of jumping back is, the more likely the random walk is to be a BFS. In contrast, parameter *q* determines the likelihood of a DFS. The skip probability of Node2Vec can be represented as (3):

$$\pi_{vx}=\alpha_{pq}(t,x)∙w_{vx}$$ (3)

However, applying 2vec model causes the loss of link meaning between nodes for heterogenous networks. Thus, in the field of KG representation, trans series are mostly used to embed entities and relations.

## 3 KG2Vec

### 3.1 Problem definitions

The walk strategy highlights the importance of nodes. If we extract the relation features between nodes, the interpretation of the node embedding can be improved. Besides, we also introduce the importance degree of nodes, improving the accuracy of embedding. In this section, we first present the reconstruction of homogeneous networks, and then the improved walk strategy.

First, we introduce some common symbols: a traditional homogeneous network includes entities, semantics, contents, attributes, relations, etc., where the first four factors constitute the nodes in the KG and the last factor is represented as the links among those nodes. A homogeneous network is expressed by triples G = (E, R, S), where *E* represents the set of entities that are nodes in the graph where E = {e1, e2, e3……,e|E|}; *R* represents the set of relations that are represented as edges where R = {r1, r2, r3……,r|R|}; and *S* represents the triples “entity-relation-entity” where S∈E×R×E, meaning that an edge links two nodes. The homogeneous networks are homogenized by reconstructing the original *G* into G’ = (E,R,W), where E represents the set of entities as in *G*, *R* represents the set of relation nodes that are extracted from the original *R* in *G*, and *W* represents the links weight either between an entity node and relation nodes or between entity nodes.

### 3.2 Heterogeneous network reconstruction

As mentioned above, the link between nodes has the same type and meaning, thus the walk process can be interpreted. However, as a kind of heterogeneous network, the walk process has to be entitled with real meanings in order to get the importance of nodes and relations.

To unify the meaning of links, we need to reconstruct the heterogeneous network. To be specific, the reconstruction process is transforming the triples into three tuples. That means, we treat a link also as a node, so that in the original homogeneous networks, one link can be separated into three to form a triangle, namely a link between entities, a link between an entity and a relation, a link between a relation and an entity. One thing to notice is that these links without any real meaning, record only the frequency of the original link. Now we define the reconstruction of heterogeneous networks.

There are two types of nodes in the reconstructed heterogeneous network G’: relation nodes and entity nodes. No link is presented between relation nodes, while ordinary links are presented between relation nodes and entity nodes. There might be links between entity nodes.

**Fig 4:**
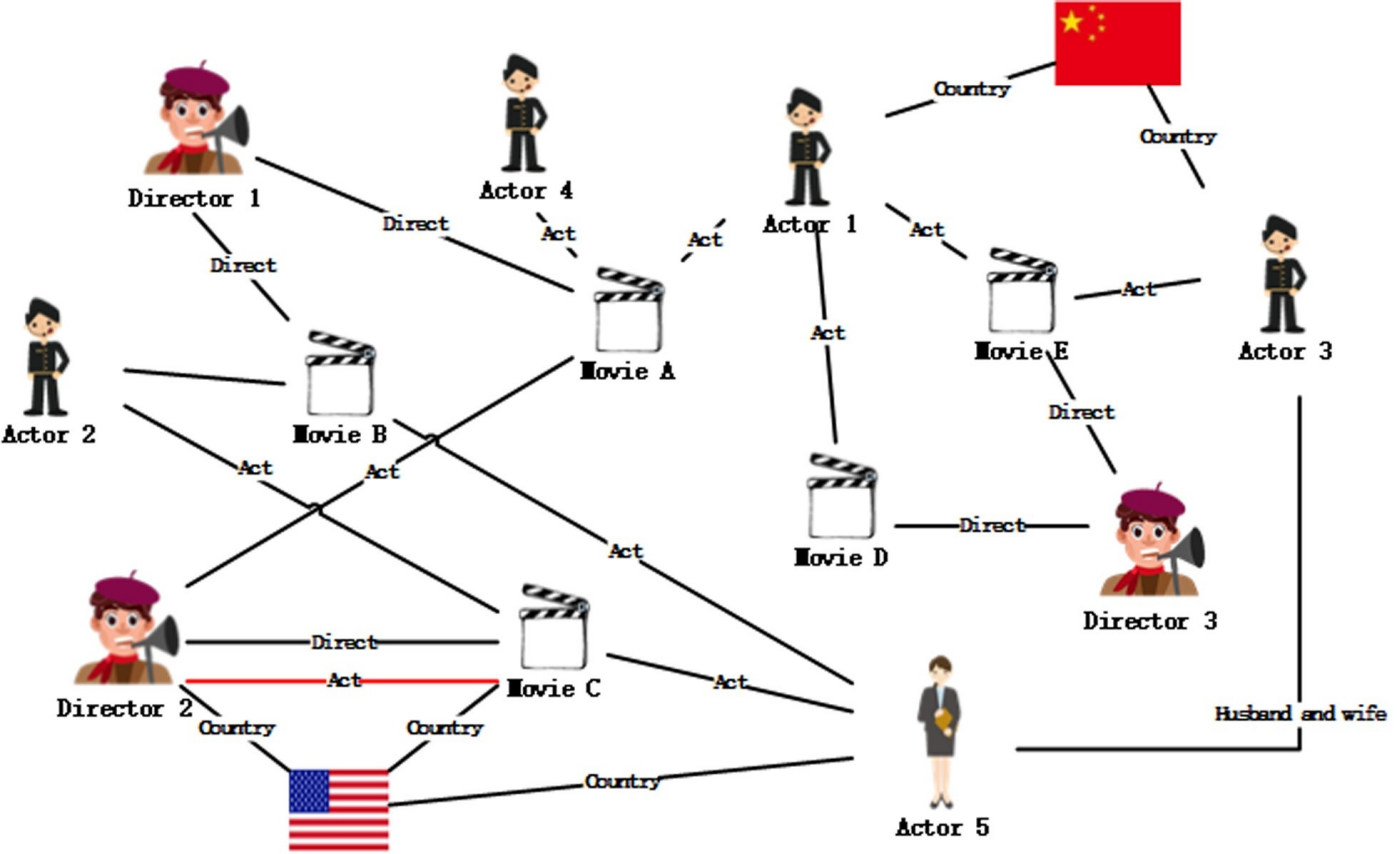
A knowledge graph of user-movies.

**Fig 5:**
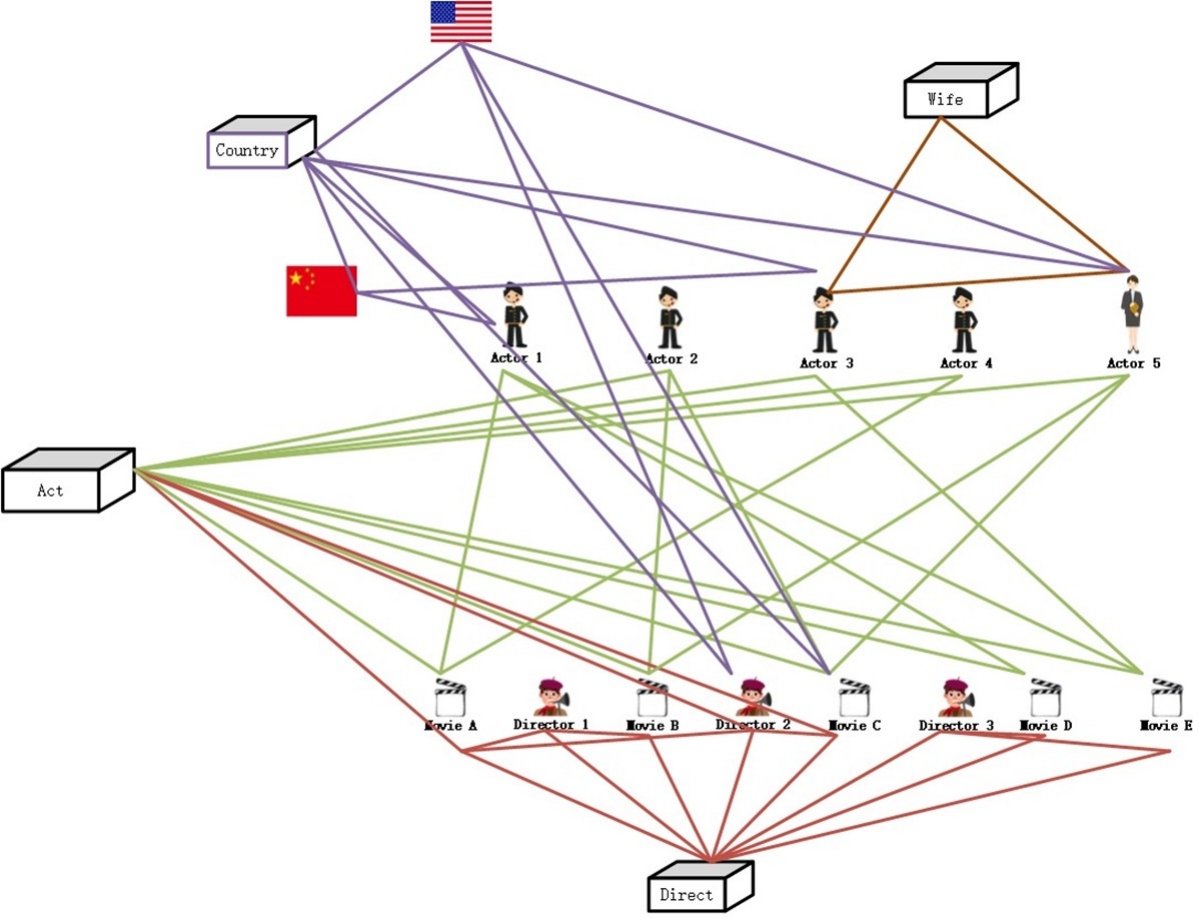
A simple knowledge graph of user-movies1.

After reconstructing Fig 4, the structure is obtained in Fig 5.

**Fig 6:**
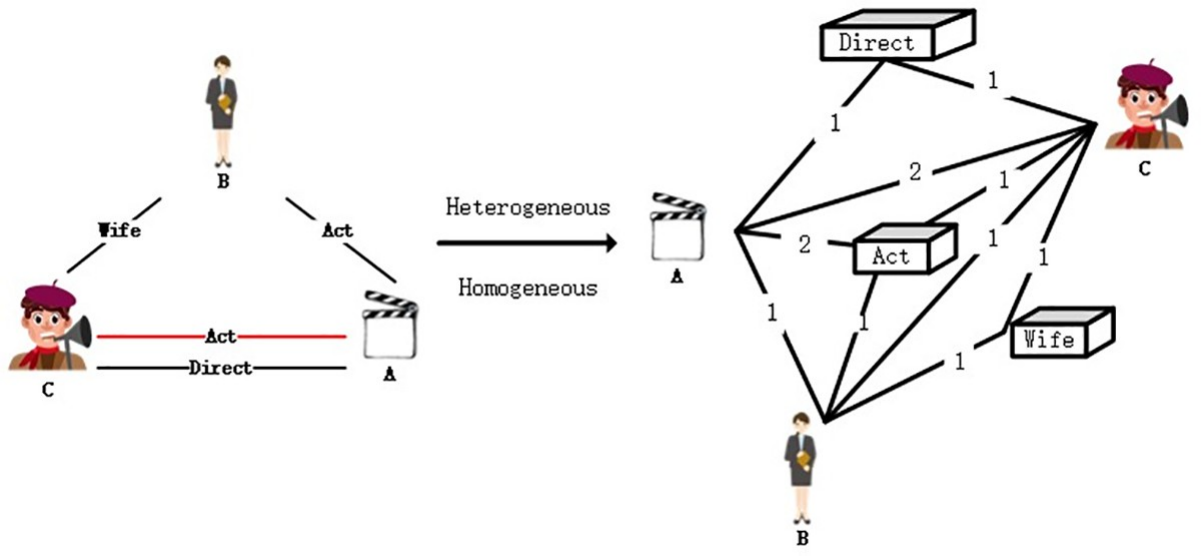
Reconstructed knowledge graph.

In order to better illustrate the process of network reconfiguration, a simple knowledge graph is used to show the reconfiguration process. Fig 6 left shows a traditional structure of the heterogeneous network: *A* is the movie title, *B* and *C* are the actors, *C* is also the director, *B* and *C* has a conjugal relationship, *D* scores *C* is *3*.

The traditional representation of heterogeneous network (such as knowledge graph) as triples (h,t,r) is:

T={(C,A,director),(C,A,actor),(C,B,wife),(B,A,actor),(D,A,3)}.

After the reconstruction, the heterogeneous network (such as knowledge graph) is represented in Fig 6 right as:

T’={(C,A),(C,director),(director,A),(C,A),(C,actor),(A,actor),(C,wife),(C,B),(B,wife),(B,Actor),(A,actor),(B,A),(D,3),(3,A),(D,A)}

As shown in the right part of Fig 6, the reconstructed heterogeneous network (such as knowledge graph) is a homogenous network.

We can see that in the original triple, we cannot identify the different importance of entity *B* and *C* to entity *A*. However, after the dissemblance of the entity and the relation, the entity pair (C,A) appears more frequently than the pair (C,B), highlighting the influence weight of entity *C* to *A*. Therefore, we entitle the links in the reconstructed homogenous network with weights, which is the frequency of the entity pair.

T’={(C,A,2),(C,director,1),(director,A,1),(C,actor,1),(A,actor,2),(C,wife,1),(C,B,1),(B,wife,1),(B,Actor,1),(B,A,1),(D,3,1),(3,A,1),(D,A,1)}

### 3.3 Walk strategy

Since the links in the homogenous network are endowed with the same meaning, sequence walks can be used to simulate the importance of nodes. However, common homogenous network only has one type of node, while the reconstructed network has two type of nodes, namely entity node and relation node. Thus, we need to alter the walk strategy as well. Compared with normal social network, KG has a special pattern that nodes exist with a sequence “entity-relation-entity”. We can set the new walk strategy according to this pattern.

In the Node2Vec model, a parameter *α* is employed to balance DFS and BFS. In this paper, this idea is inherited in the proposed KG2Vec model, yet altering the setting of walking parameters *p* and *q*. Now, different random walks are proposed for different types of nodes.

#### 3.3.1 Entity nodes

**Fig 7:**
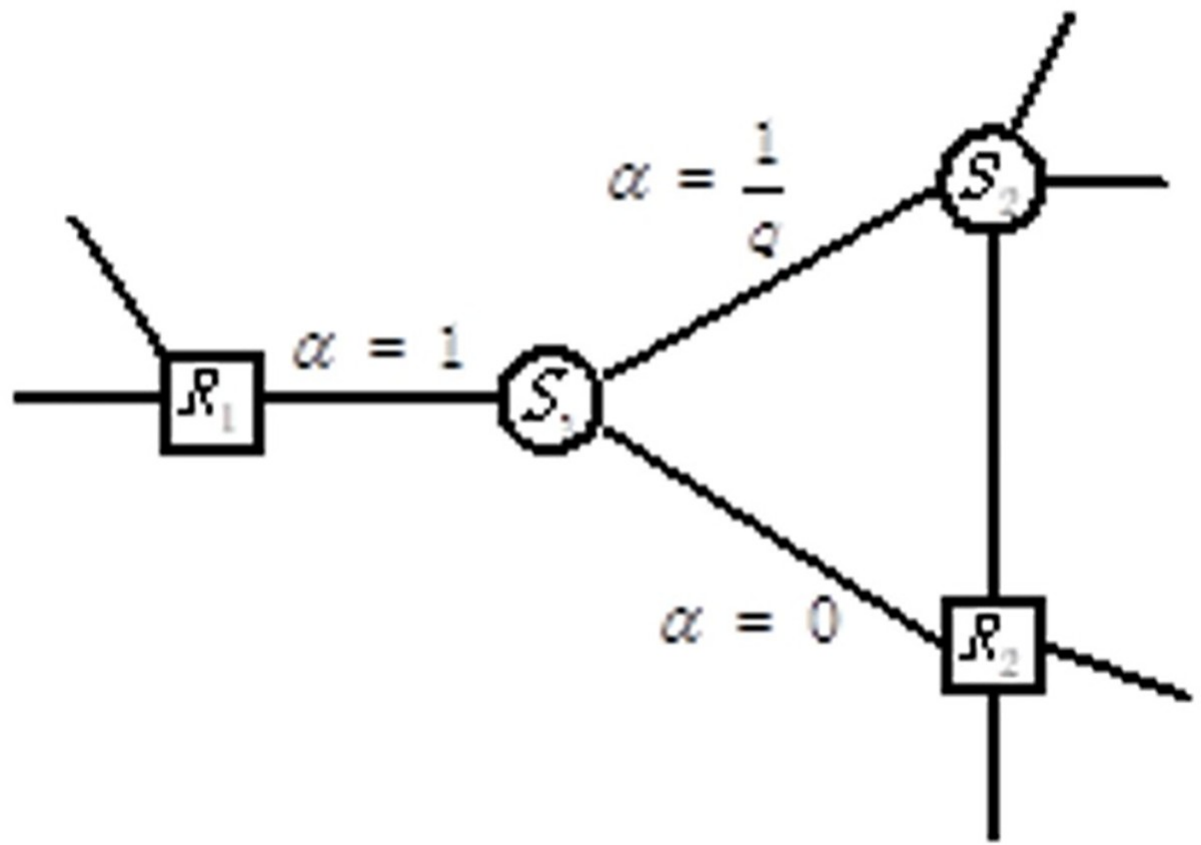
Entity node transition probability.

As shown in Fig 7, it is supposed that the current node is the entity node S1, which can be reached from the relation node R2. As the next jump transition, there are three situations that could happen. The corresponding probabilities are as follows.

- Jumping back to the last relation node *R2* is meaningless for analysing a heterogeneous network, and hence the probability is 0.
- Jumping to another connected relation node *R1* is exactly what we want, as illustrated in hypothesis 1, and hence, the probability is 1.
- Jumping to another connected entity node *S2* is abnormal, and hence, the probability is set to *1/q*.

The skip parameter *α* of entity nodes is summarized as Eq 4:

$$\alpha_{q}(t,x)=\left\{ \begin{array}{c} 0\;if\;d_{tx}=0\\ \frac{1}{q}if\;d_{tx}=1\\ 1\;if\;d_{tx}=2 \end{array}\right.$$ (4)

where *α* is the skip parameter; *q* is the training parameter; *dtx* represents the shortest path between nodes t and x, which satisfies second-order Markov model; and the value of *dtx* is located in the set of {0,1,2}.

#### 3.3.2 Relation nodes

**Fig 8:**
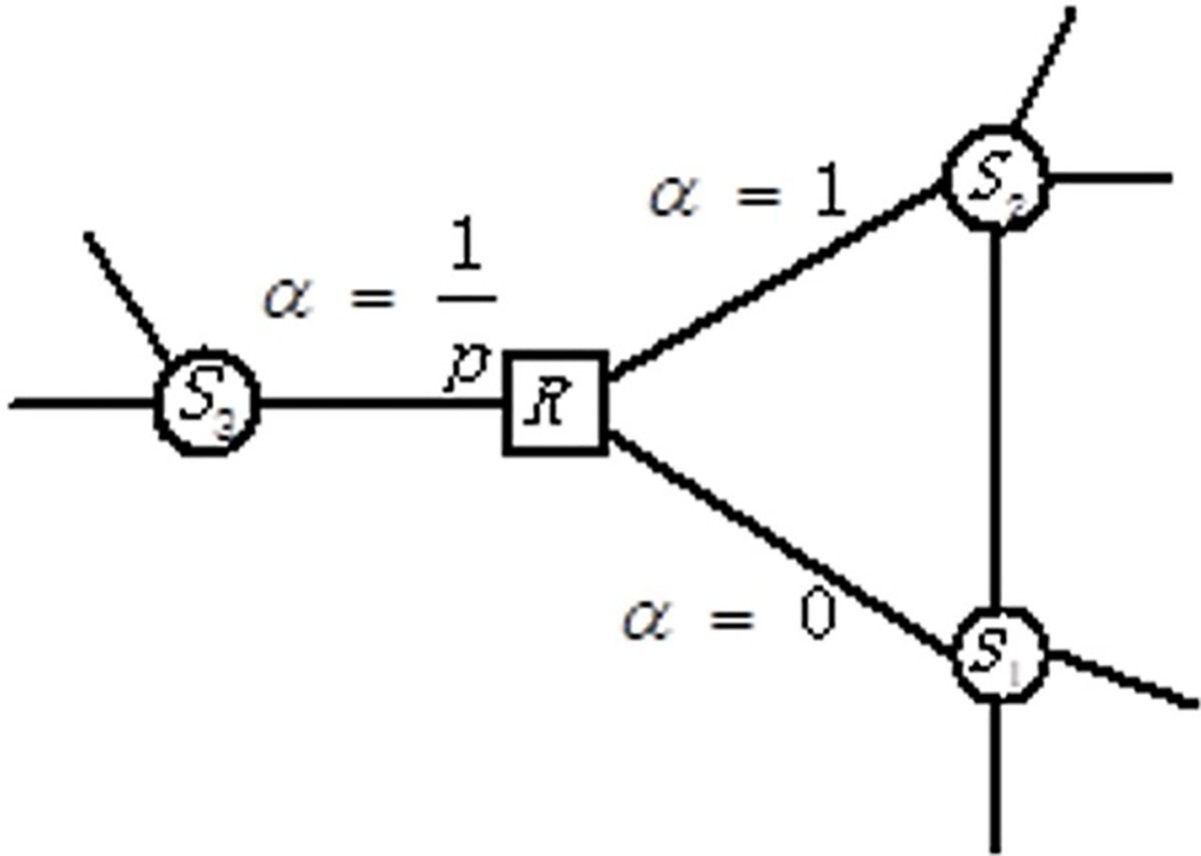
Relation node transition probability.

Supposing the current node belongs to the relation type and, according to Hypothesis 1, relation nodes can only be connected to entity nodes. As shown in Fig 8, the current node is *R*, and the last node is *S1*. The next transition of node R includes three situations.

- Jumping back to the last node *S1* does not exist in the heterogeneous network, and hence the probability is 0.
- Jumping to the node *S2* that is connected with the last node S1 constitutes the logical sequence “entity-relation-entity”, which is exactly as expected in hypothesis 1, and hence the probability 1.
- Jumping to another connected entity node S3 constitutes the logical sequence “entity-relation” and “relation-entity” which could happen but is unexpected and hence the probability is set to *1/p*.

The skip parameter α of the relation nodes is summarized as Eq 5:

$$\alpha_{p}(t,x)=\left\{ \begin{array}{c} 0\;if\;d_{tx}=0\\ 1\;if\;d_{tx}=1\\ \frac{1}{p}if\;d_{tx}=2 \end{array}\right.$$ (5)

where *α* is the skip parameter, p is the training parameter, *dtx* represents the shortest path between nodes *t* and *x* satisfying the second-order Markov model, and the value of *dtx* is located in the set {0,1,2}.

The skip probability of KG2Vec can be represented as Eq 6:

$$\pi_{vx}=\alpha_{pq}(t,x)∙w_{vx}$$ (6)

### 3.4 Training model

Originally, Node2vec was inspired by Word2vec, which integrates NLP into social networks. Moreover, KG2Vec, which is based on Node2vec, deals especially with a heterogeneous network, which is a complex HIN. In comparison, Node2vec, which was proposed by Aditya, uses skip-gram for training. While the Word2vec training method is divided into the CBOW and skip-gram. Our KG2Vec combines those two methods to achieve better performance.

Additionally, there are two purposes of Word2vec: one is to predict the centre word given context, and the other is to predict the context given the centre word [29]. For the first situation, the CBOW is applied to perform the prediction, and skip-gram is applied for the second situation. However, in a heterogeneous network, the input sequence should obey the mode of “entity-relation-entity”.

**Fig 9:**
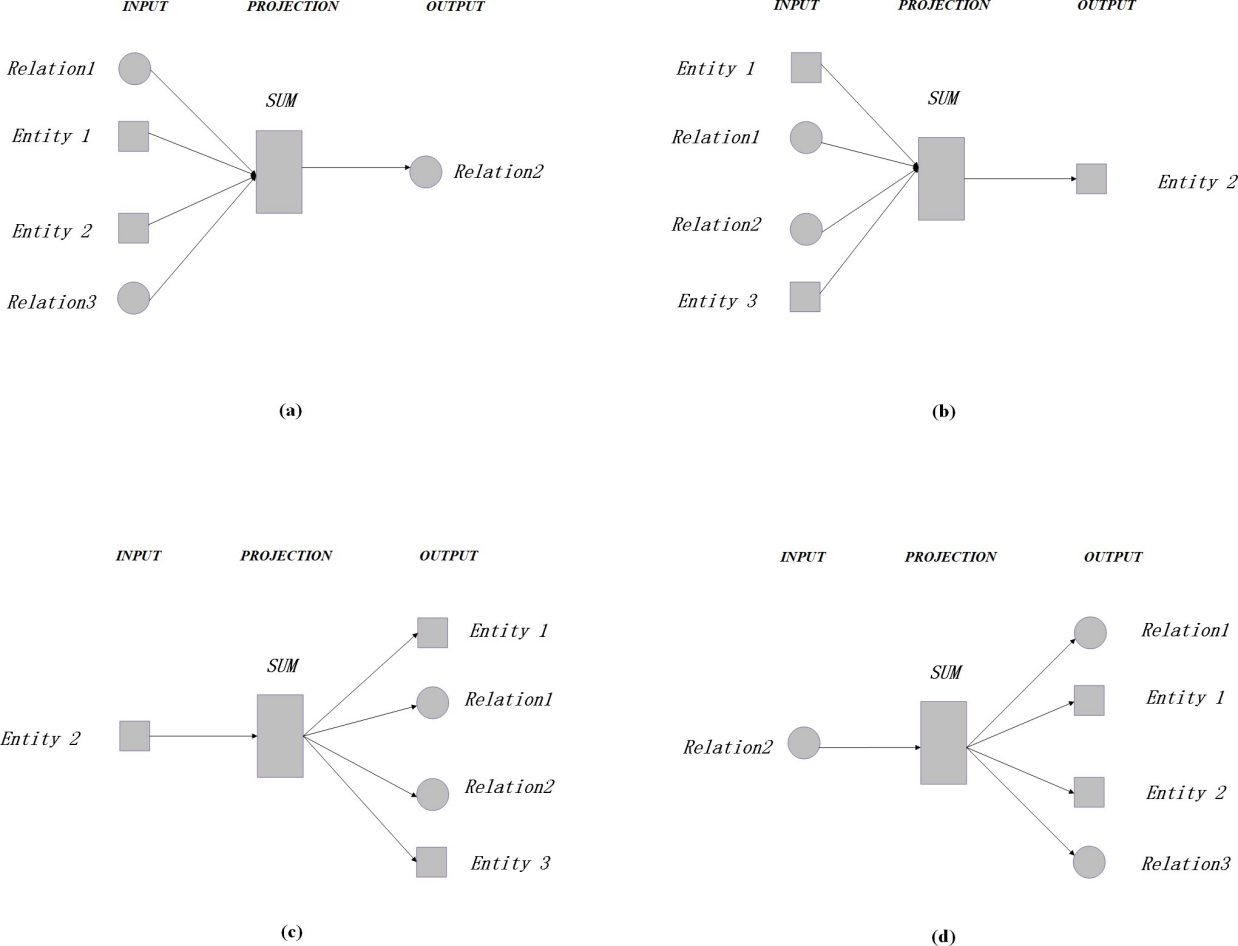
The KG2Vec model (a. the prediction of the relation using the CBOW, b. the prediction of the entity using the CBOW, c. the prediction of the entity’s context using Skip-gram, and d. the prediction of the relation’s context with Skip-gram).

Similarly, we could use different algorithms under different circumstances. For those relation nodes, the skip-gram is applied to predict the entity nodes of the context of the sgiven relation nodes and CBOW is applied to predict the relation nodes given the context of entity nodes. The same strategy is used for entity nodes to predict context and centre words. The following Fig 9 shows the prediction algorithm of KG2Vec for entity nodes and relation nodes.

### 3.5 Optimizing random walks

To improve the accuracy and efficiency of the random walks, a parameter that expresses the influence of nodes is proposed. In Node2vec, no such parameter is taken into account, and it treats every node as equivalent, consequently lowering the quality of the sample data.

Therefore, the node degree is defined as the influence parameter. That is, nodes with higher influence would have more walks, and contrarily, nodes with lower influence would have fewer walks [30]. Additionally, a threshold is introduced to limit the upper bound of the walks. That is, when the node degree reaches this threshold, the maximum number of walks is used to train the model, and when the node degree is below this threshold, the number of walks is reduced according to the proportion of the influence. In conclusion, we define the walks *Np* for node *p* as Eq 7:

$$N_{p}=\left\{ \begin{array}{c} N_{max}\times \frac{D_{p}}{D_{max}}D_{p}<t\\ N_{max}\;D_{p}\geq t \end{array}\right.$$ (7)

where *N*_*max*_ is the maximum number of walks, *D*_*p*_ is the degree of node *p*, *D*_*max*_ is the maximum degree among all the nodes, and *t* is the threshold.

Algorithm 1 is the pseudo code of KG2Vec. First, the walks *N*_*max*_ are updated according to each node degree. Then, the learning process is optimized by simulating the random walk with an *N*_*max*_ of length l from each node *u*, which neutralizes the implicit bias, and by calculating the transition probability, which complements the walking sequence as the selection of node context. Then, according to the type of the current node, which is either an entity node or relation node, we choose an appropriate random walk strategy as discussed before. After the context is obtained, we apply the SGD to simulate random walks and optimize the process. Thus, the algorithm is shown as follows.

Algorithm1 The KG2Vec algorithm

LearnFeatures (***G*** = ***E*,*R*,*L***), Dimensions ***d***,

MaxWalks ***N***_***max***_, Walk length l, Context size ***k***,

thresholdValue ***t***, Return ***p***, In-out ***q***)

***π* = *PreprocessModifiedWeights* (*G*,*p*,*q*)**

***G*′ = (*E*,*R*,*L*,*π*)**

Initialize walks to Empty

***If*** *nodeDegree***<*t***

    ${N_{max}\leftarrow N}_{max}\times \frac{D_{p}}{D_{max}}$

***end if***

***for*** iter **=** 1 to ***N***_***max***_ do

        ***for*** all nodes ***u***∈***E*** or ***u***∈***R*** do

            ***walk*←**KG2Vec**(*G*’,*u*,*l*)**

            Append walk to walks

        ***end for***

***end for***

                    ***f* = *StochasticGradientDescent*(*k*,*d*,*walks*)**

  ***return f***

KG2Vec (KGraph ***G***’ = (***E*,*R*,*L*,*π***),Start node ***u***, Length ***l***)

Initialize ***walk*** to ***u***

***For walk_iter* =** 1 to l do

    curr ←walk [−1]

    *if* ***u***∈***E***

        V_curr_←GetEntiyNeighbors (curr,G’)

    ***else***

        V_curr_←GetRelationNeighbors (curr,G’)

    ***end if***

    s← AliasSample (V_curr_,Π)

Append ***s*** to walk

***end for***

    ***return walk***

## 4 Experiment

### 4.1 Data set

Two data sets, which are common KG data: WordNet and Freebase, are used. WordNet is a lexical database. In WordNet, an entity is composed of one or several words, forming a synset. A single word can belong to different synsets. The relation between synset includes hypernym, hyponym, mer, hol, and troponym relationships. Freebase is a large collaborative knowledge graph that contains common world facts. WN18 [31] and FB15K-237 [32] are employed in Wordnet to evaluate the Recall. In addition, WN18 dataset is employed to evaluate the link prediction meanRank value.

Table 1 shows the basic parameters and settings of the above five data sets.

**Table 1:** Data sets.

	ENTITY	RELATION	TRIPLE
FB15K	14541	41	272115
WN18	40943	18	86835

KG2Vec is compared with the following baseline. One thing to mention is that the parameter settings remain the same with the original paper unless otherwise specified.

**TransE**: As mentioned above, this algorithm represents KG as triples and uses iterative training to minimize the value of h+r−t until convergence. Experimental parameters are set as follows: margin = 1, learningRate = 0.00001, dim = 8, L1 = True.

**Node2Vec**: Node2Vec is one of the bases of our proposed algorithm. We set the parameters p = 4, and q = 1(the same as original paper), workers = 8, embedding_dim = 8, walking_length = 80, num_walks = 10.

### 4.2 KG2Vec parameter tuning

The KG2Vec trains the vector representations of entities and relations, which should be equipped with all the properties of other vectors (e.g., word vectors). One of the most important properties is similarity. For instance, word vectors could reveal the semantic similarity between two entities or words, thereby implying that those semantically similar entities should be closer in space, and further apart otherwise. Therefore, the similarity is adopted as an evaluation criterion. The similarity of entities and relations are separated as follows.

**Definition 1**: Similar entities are those that have the same relation node and are connected to each other. Similar relations are those who point to the same entity or those who are pointed to by the same entity. Furthermore, the recall is used as the evaluation standard. To start, all the similarities between the current entity node and other entities nodes (or the current relation node and other relation nodes) are calculated. Then, the similarities are sorted, and the proportion of similar nodes in the first N nodes is calculated as Eq 8.

$$R_{i}=\frac{N_{isim}}{N}$$ (8)

where *R*_*i*_ represents the recall of node *i*, *N* represents the top *N* after sorting the similarities, and *N*_*isim*_ represents the number of similar nodes to node *i* among those *N* nodes. To calculate the similarity between two nodes, the Euclidean distance is used as Eq 9.

$$S_{ij}=\sum_{N}^{i=1}{\left(I_{i}-J_{i}\right)}^{2}$$ (9)

where *N* represents the number of vector dimensions, *I*_*i*_ represents the *i*-th dimension of vector *I*, and Ji represents the *i-*th dimension of vector *J*.

**Fig 10:**
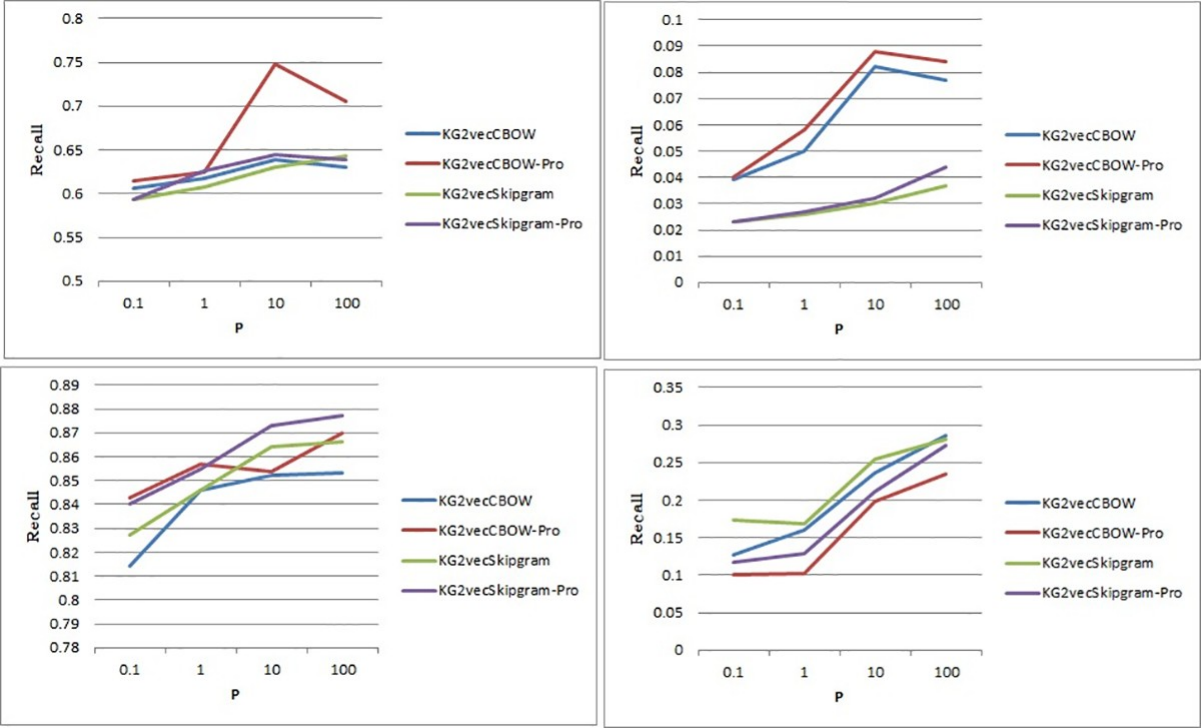
Line chart of P (a is the entity data of the FB15K data set, b is the relational data of the FB15K data set, c is the entity data of the WN18 data set, and d is the parameter relational data of the WN18 data set).

Regarding random walks, the values of parameters p and q need to be set. In KG2Vec, p is used to adjust the walks of relation nodes, while q is used to adjust the walks of entity nodes. To achieve the best performance, the values of p and q are tuned as shown in Fig 10.

It can be concluded from Fig 10 that the bigger the initial value of p is, the higher the recall is, no matter whether entity vectors or relation vectors are used. It can also be observed that when p is between 10 and 100, the recall does not differ much. Under some circumstances, p = 10 results in better performance. Therefore, 10 is used as the initial value of p. Another interesting result to note is that after introducing the optimizing training algorithm, the recall increases both with the CBOW and Skip-gram.

**Fig 11:**
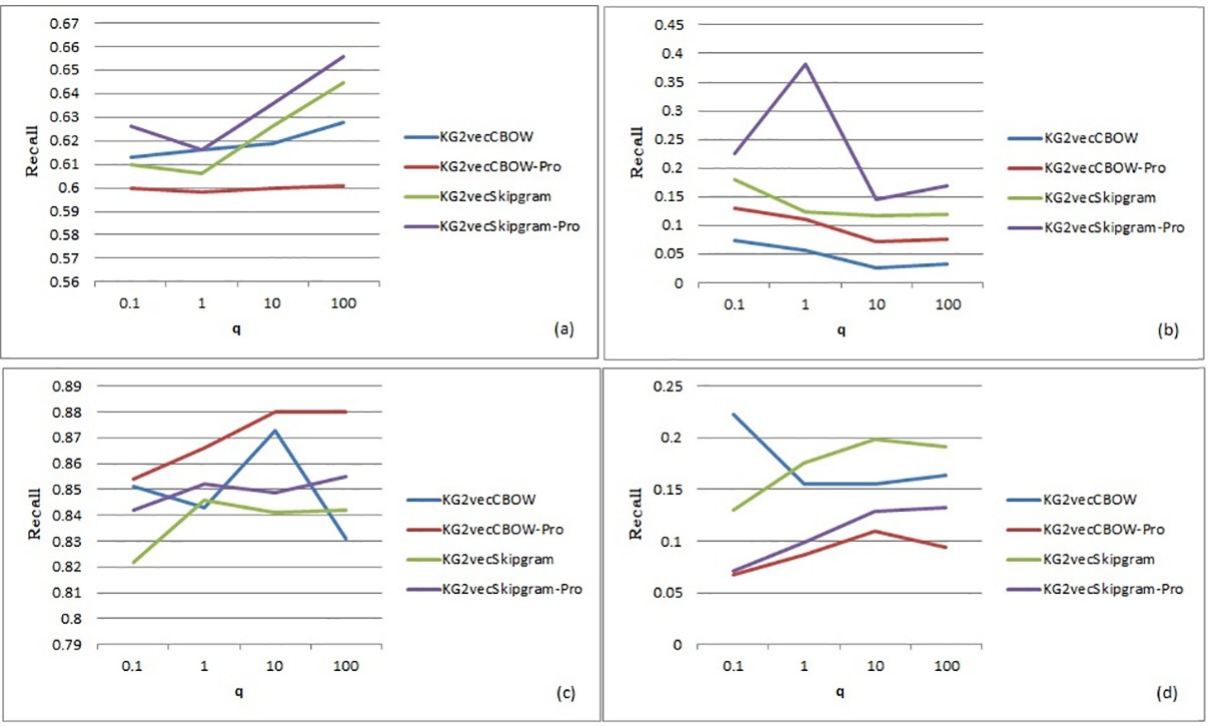
Line chart of q (a is the entity data of the FB15K data set, b is the relational data of the FB15K data set, c is the entity data of the WN18 data set, and d is the parameter relational data of the WN18 data set).

From Fig 11, it can be seen that there is no explicit regularity for q affecting the recall as p shows; nevertheless, the recall fluctuates greatly under different circumstances. This means that the effect of q on the walks is random. Therefore, q = 100 is finally chosen as the initial value the recall has the tendency to increase as q increases (C. Shi *et al*,2016) [17]. Furthermore, the optimized training algorithm that is illustrated above has little influence on the experimental recall-q, which also proves that in our proposed random walks, p has more influence than q.

### 4.3 Result

The experiment results of recall on FB15K-237 are shown in Table 2. It can be seen that the algorithms with knowledge representation learning based on random walks, regardless of whether Node2Vec or KG2Vec is used, has better performance than those with space shifting, such as TransE. This means that the knowledge representation learning based on random walks results in more semantically sufficient representation vectors. Furthermore, our KG2Vec outperforms Node2Vec, both in entity vector learning and relation vector learning. Another observation is that the CBOW performs better than Skip-gram during vector learning, which may be because the CBOW predicts the current entity/relation based on context and Skip-gram uses the entity (or relationship) to predict the entities and relationships around it. In particular, there are not many nodes around the entity node that are connected to it, so they can’t travel route to its entity (or relationship). It can be also found that the proposed optimization algorithm in CBOW model training and Skip-gram has achieved good ascension. This shows that our training optimization algorithm can solve the problem of model overfitting caused by the high noise and low quality of data caused by the power law property of complex networks. As knowledge graph is also a kind of social network, power law problems can also appear in other social networks. Therefore, the training optimization algorithm proposed in this paper has strong universality and can be applied to solve other social network problems.

**Table 2:** Experimental results of KG2Vec model on FB15K-237.

MODEL	ENTITY	RELATION
Node2Vec	0.608	0.019
KG2Vec (cbow)	0.621	0.034
KG2Vec(cbow+improved)	0.616	0.054
KG2Vec (skip-gram)	0.613	0.021
KG2Vec(skip-gram+improved)	0.615	0.048
TransE (baseline)	0.497	0.022

The experimental result of recallon WN18 is shown in Table 3. We can see the same result as that for FB15K-237. Both Node2Vec and KG2Vec that use knowledge representation learning based on random walks outperform those that use space shifting, such as TransE. Additionally, KG2Vec improves the vector learning of both entities and relations compared to Node2Vec. Therefore, it can be concluded that KG2Vec, which applies knowledge representation based on random walks and is tailored for knowledge graphs, achieves the best performance since it considers the full semantical characteristics of both entities and relations.

**Table 3:** Experimental results of KG2Vec model on WN18.

MODEL	ENTITY	RELATION
Node2Vec	0.826	0124
KG2Vec (cbow)	0.846	0.127
KG2Vec(cbow+improved)	0.849	0.106
KG2Vec (skip-gram)	0.844	0.166
KG2Vec(skip-gram+improved)	0.843	0.139
TransE (baseline)	0.697	0.101
Node2Vec	0.826	0.124

In conclusion, our experiments show that the algorithm that learns the knowledge representation based on random walks can obtain a better representation vector, which takes full advantage of learning the semantics of entities and relations, compared to TransE. TransE is an algorithm that is based on the shifting of the space vector, which is a shallow algorithm. Deeper algorithms, such as the neural network based Node2Vec and our proposed KG2Vec, result in better semantic expressed vectors, which has been proven by this paper and others.

The CBOW model and Skip-gram model in KG2Vec have little difference in the learning of entity representation vector, but differ in the learning of relation representation vector, which is slightly different from the experimental results on the FB15K data set. Compared with CBOW model, the relationship representation vector learned by Skip-gram model is better. This may indicate that Skip-gram model of KG2Vec is a better method in relation representation vector. In addition, it is worth noting that the training optimization algorithm proposed in this paper still has a good effect, which further illustrates the superiority of the training optimization algorithm.

Two algorithms are also proposed with the CBOW and Skip-gram. The experimental results indicate that both algorithms equally improve the KG2Vec, more specifically, the CBOW performs better when learning entity representation vectors and Skip-gram is more suitable for learning relation representation vectors.

By summarizing the experimental results on two data sets, it can be found that compared with the model represented by TransE, the knowledge representation learning model based on random walk can fully learn the semantics of entities and relationships. This is because:

1. Knowledge representation based on the random walk model aims at learning map entities (relationship) sequence in modeling, it focuses on a pair of entities and relationships, instead of focusing on the relationship between entities and relationships in order at the same time also pay attention to entities and relationships in the knowledge graph in the network, which well solves the limitation of learning knowledge representation model based on spatial translation.
2. Based on the random walk knowledge representation learning model, both node2VEc and the KG2Vec models proposed in this paper use Word2VEC model, and Word2VEC model itself is a neural network model, which is a deep model. TransE model, on the other hand, is based on vector translation in space, and it is a shallow model.

CBOW model is better at learning entity representation direction, while Skip-gram model is more suitable for learning relational representation vector, which may be related to the principles of CBOW and Skip-gram models.

Finally, it can also be seen that both FB15K and WN18 models have lower recall rates in relation vectors, which may be caused by the following two reasons:

1. In the knowledge graph, the number of relational nodes is smaller than the number of entity nodes. Both the traditional knowledge representation learning model and random walk need a large number of training samples to improve the training effect of the model and avoid the problem of under fitting and overfitting. Therefore, it is not surprising that the experimental results do not show the ideal situation when the number of relational nodes is small.
2. In the previous definition of the concept of relational similarity, we mentioned that the definition of similar relationship is vaguer than that of similar entity, so it is difficult to ensure that the definition can accurately identify similar relationship.

In any case, the KG2Vec model proposed in this paper still performs better than the traditional knowledge representation learning model. From this point, it can still prove the superiority of knowledge representation learning based on random walk.

## 5 Conclusion

An improved knowledge representation learning algorithm that is based on a modified random walk called KG2Vec is proposed, which is inspired by Node2Vec but tailored to KGs. The algorithm regards relations as the nodes in the network and reconstructs KGs, yielding two types of nodes: entity nodes and relation nodes. Afterwards, the algorithm utilizes the strategy in Node2Vec to generate node sequences, which is then further trained using Word2vec. Moreover, experiments on the FB15K237 and WN18 KG databases are conducted and the entity and the relation nodes to obtain their representation vectors. The results of recall shows that KG2Vec is efficient and effective on real-world data. However, our model has a high computational complexity, and time cost arising from representing each triple separately. How to improve the efficiency of the feature extraction is an urgent problem to be solved. In addition, the temporal and spatial attributes for dynamic KGs also need to be studied as the next research goal.
